# “Paint and Sip for Cancer Prevention”: A Novel Arts-Based Community Engagement Strategy to Advance Cancer Education and Screening in Underserved Individuals

**DOI:** 10.1007/s13187-025-02679-y

**Published:** 2025-06-28

**Authors:** Lisa Carter-Bawa, Francis Valenzona, Miriam Lucca-Susana, Gary Kwok, Elan N. Shoulders

**Affiliations:** 1 Cancer Prevention Precision Control Institute, Hackensack Meridian Health Center for Discovery & Innovation, 123 Metro Blvd, 6th Floor, 6400 Pod, Nutley, NJ 07110 USA; 2 Hackensack Meridian School of Medicine, Nutley, NJ USA; 3 Georgetown Lombardi Comprehensive Cancer Center, Washington, DC USA; 4 John Theurer Cancer Center, Hackensack Meridian Health, Hackensack, NJ USA

**Keywords:** Cancer prevention, Cancer control, Arts-based intervention, Community outreach, Community engagement

## Abstract

Persistent disparities in cancer awareness, screening uptake, and early detection disproportionately affect underserved communities in the USA. While traditional health education approaches play a critical role, they often fail to engage historically marginalized populations in culturally resonant ways. Innovative strategies that leverage creativity, trust-building, and community participation are urgently needed. In 2024, the Community Outreach and Engagement (COE) Department at the John Theurer Cancer Center launched “Paint and Sip,” an arts-based cancer education series designed to increase engagement and awareness in diverse communities. Events were held in accessible, non-clinical venues and combined guided painting sessions with culturally tailored cancer education, delivered by trained community health workers (CHWs). During natural pauses in the painting process, CHWs led 15-min discussions on cancer prevention topics, including screening guidelines, risk factors, and genetic testing. All participants completed a brief social determinants of health screening questionnaire and were offered patient navigation services to address unmet needs and support screening follow-through. Since its launch, the series engaged 191 participants across multiple community settings. Attendance averaged 25 per event, with strong retention and word-of-mouth promotion. Among participants, 82% were not up to date with cancer screening, and 62% accepted navigation support. Qualitative feedback highlighted the events as “uplifting,” “judgment-free,” and “a new way to learn about health without fear.” CHWs reported increased participant openness and discussion around stigmatized topics. The Paint and Sip model illustrates the promise of arts-based engagement as a novel strategy for advancing cancer education and screening in underserved communities. By embedding creativity into health education, the model fosters trust, enhances message retention, and bridges awareness with action. Further research is warranted to evaluate long-term impact, scalability, and cost-effectiveness.

**Supplementary Information:**

The online version contains supplementary material available at 10.1007/s13187-025-02679-y.

## Introduction

Despite decades of progress in cancer prevention and control, persistent disparities in awareness, screening, and early detection remain in underserved US communities [1]. Community outreach and engagement has emerged as a key strategy for addressing these gaps, especially within National Cancer Institute (NCI)-designated cancer centers [2, 3]. Yet, *how* to meaningfully engage historically marginalized and underserved populations remains complex.

Traditional health education methods often fall short in building trust and fostering sustained engagement [4, 5]. Increasingly, creative and culturally responsive, arts-based strategies are being recognized as effective tools for engaging communities, enhancing awareness, and catalyzing behavior change [6]. Artistic expression offers more than aesthetic value—it opens a pathway to emotional connection and shared learning in psychologically safe spaces. Used intentionally, these approaches have the potential to humanize public health messaging, reduce stigma, and activate community strengths toward health equity [7].

This paper explores how structured, arts-based programming can advance cancer education and prevention. We highlight a 2024 initiative from the John Theurer Cancer Center’s Community Outreach & Engagement (JTCC-COE) Program: a series of “Paint and Sip” events combining guided artistic expression with tailored health education, community health worker (CHW) engagement, and linkage to services. This model illustrates how creativity can foster participatory action, reduce screening barriers, and build trustworthiness between a health system and its community.

## Creativity and Community Engagement: A Framework for Action

Community outreach and engagement (COE) is fundamentally about relationship-building and mutual learning [2]. Traditional top-down health education often falls short, particularly in communities that have experienced medical mistrust or systemic barriers [2]. Emerging evidence underscores the importance of participatory approaches that center community voices and cultural relevance [6–8].

Arts-based engagement draws from community-based participatory research (CBPR), adult learning theory, health communication, and the arts in health movement. CBPR emphasizes co-learning and shared power; when designed with community input, arts programs invite inclusive participation across cultural, linguistic, and educational backgrounds [9]. Within this framework, creativity is not a luxury but a tool for equity—offering novel ways to communicate, collaborate, and co-create knowledge. Arts-based engagement programs, when designed with community input, allow for inclusive participation and resonate across linguistic, educational, and cultural boundaries. Importantly, they reflect CBPR’s principle of meeting communities *where they are*, not just geographically, but also emotionally and culturally [9].

Adult learning theory supports integrating creative modalities into health education [10]. Adults engage more when learning is experiential, relevant, and delivered in respectful environments [10]. Art-based activities, like painting or storytelling, facilitate reflection and internalization of health concepts. From a health communication lens, creativity boosts message salience and emotional resonance [11]. Visual imagery and metaphor can make abstract or stigmatized topics, like cancer risk, more accessible [11]. Art can reduce resistance and enable open discussion of personal or cultural beliefs.

The arts in health movement further demonstrates how creative expression improves health outcomes, mental well-being, and community connection [12, 13]. In cancer prevention and control specifically, arts-based interventions have been used to reduce stigma, increase knowledge, and promote dialogue around sensitive topics such as cervical cancer, breast health, or colorectal cancer screening [8, 14, 15]. These interventions often succeed not only because of the information they convey, but because of the context in which they are delivered—a context shaped by creativity, emotional safety, and shared experience. Taken together, these frameworks and findings suggest that creativity is not peripheral to community engagement; rather, it is essential. Creativity-based strategies can help transform outreach events from transactional moments into transformational experiences that build trust, encourage dialogue, and ultimately move individuals toward informed action. This program had three aims: (1) to engage underserved community members through an arts-based format; (2) to raise awareness and support informed decisions about cancer screening and related early detection modalities such as hereditary cancer genetic testing; and (3) to connect participants with navigation services that convert awareness into action.

## Methods

### Program Design and Setting: One Cancer Center’s Model: Creativity in Action

**Fig. 1:**
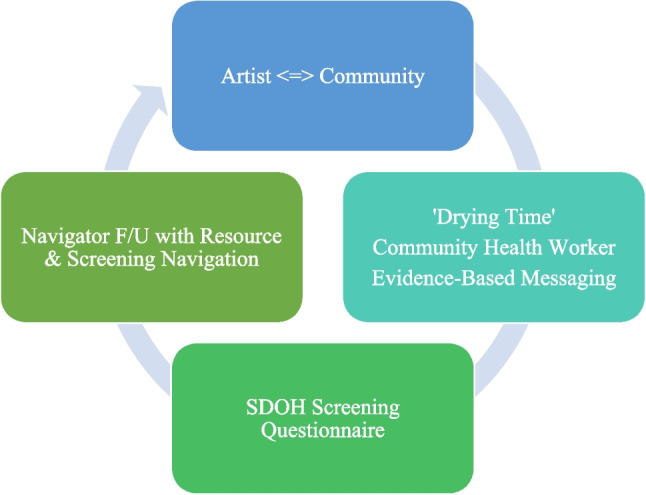
The ‘Paint and Sip’ model

In 2024, the JTCC-COE Department launched “Paint and Sip,” an arts-based series designed to engage communities around cancer prevention and early detection. This creative initiative was developed in response to a recurring challenge faced by many COE programs—how to foster meaningful engagement on cancer-related topics in a way that feels welcoming, inclusive, and culturally resonant. The result was a hybrid model that blends art, social connection, and evidence-based education in accessible, non-clinical spaces (see Fig. 1).

Paint and Sip events were held in accessible local venues such as libraries, community centers, or houses of worship, where participants were warmly greeted with non-alcoholic “mocktails” and light refreshments. Events were free and open to all adults, with bilingual facilitation where appropriate. A professional artist guided attendees through the creation of a painting thematically aligned with cancer education topics, such as:

- A representational image of *Pearls of Wisdom* for colorectal cancer awareness
- *Ornaments* symbolizing genetic connection for a presentation about genetic testing services
- A *tree of life* symbolizing women’s health

## Participant Recruitment

Paint and Sip events were promoted by community-based partners, flyers in high-traffic areas, word of mouth, and social media.

## Session Structure and Content

Events were capped at 40 participants to preserve an interactive atmosphere. Each session combined artistic expression and cancer education. CHWs use these art pieces (e.g., Pearl of wisdom imagery for colorectal cancer awareness, ornaments symbolizing genetic connection, tree of life for women’s health) as conversation starters, discussing personal experiences and cancer-related beliefs. A key innovation is the intentional use of “drying time”—the natural pause in the painting process—for a 15-min health education session led by CHWs tailored to the event’s theme. “Drying time” refers to the time interval needed for the wet acrylic background layer to air-dry so the next coat will not mix into the background layer. Rather than letting this natural pause go unused, CHWs deliver an interactive cancer education mini-lesson during the interval, turning idle wait time into purposeful learning and discussion that directly supports the program’s cancer prevention and control goals. Paint and Sip sessions lasted 120 min and followed a standardized, artist-led script (see Supplemental) co-created with the artist and the JTCC-COE team. The flow was as follows: (1) welcome, gratitude, and a brief grounding exercise (~ 2 min); (2) introduction of the artist, CHWs, and patient navigator (~ 3 min); (3) “tools of the trade” demonstration, introduction to the steps of the painting process, and affirmation that “there is no wrong mark” (~ 5 min); (4) phase 1 painting – white wash and background color/pattern (~ 20 min); (5) 15-min ‘drying-time’ cancer education presentation and dialogue led by a CHW; (6) phase 2 painting—foreground figure/embellishments with small brush, peer encouragement, and sharing of a personal cancer story (~ 35 min); (7) closing reflection, pledge, and resource table visit (~ 10 min); and (8) clean-up (~ 30 min). Light refreshments, including non-alcoholic “mocktails,” help to reinforce a relaxed, judgment-free atmosphere. See Supplementary Material for a detail of the event protocol.

Informed by feedback obtained from the JTCC Community Advisory Council—a council of community key stakeholders that meets quarterly to guide the programmatic initiatives of the department’s community outreach and engagement initiatives—topics included the following:

- Colorectal cancer screening guidelines and myths.
- Breast cancer risk and screening eligibility.
- Cancer risk and screen-detectable cancers and “your why” for screening.
- Genetic testing and familial risk.
- What is a clinical trial.

CHWs provided culturally tailored education and encouraged participants in dialogue. These interactions extend beyond basic information-sharing by fostering meaningful dialogue, dispelling myths, and encouraging personal storytelling in a supportive, peer-driven environment. Each event (regardless of the specific topic) also included a dedicated table for distributing cancer education materials and fecal immunochemical test (FIT) kits to risk-appropriate individuals to promote colorectal cancer screening uptake. Importantly, all participants were invited to complete a brief social determinants of health (SDOH) screening questionnaire (described in more detail below) before the conclusion of the event to identify unmet needs (e.g., housing instability, food insecurity, transportation barriers, limited healthcare access). A JTCC-COE patient navigator followed up within 48 h to connect participants with resources and screening support, ensuring that education had the potential to translate into action. By pairing creativity and art-making with supportive conversations facilitated by CHWs, participants are empowered to share personal narratives, address concerns, and overcome barriers in a non-threatening, supportive environment. This approach reduces the intimidation often associated with formal health education, promotes emotional openness, and fosters a sense of communal trust and solidarity, ultimately enhancing receptivity to cancer screening messages and health-promoting behaviors.

## Data Capture and Linkage

Because most event attendees are *not* patients in our health system electronic health record (EHR), we built a stand-alone, community-based EHR using REDCap. Each participant record creates a unique record ID for the individual upon entry into REDCap; CHWs immediately enter demographic information, cancer-risk factors, the education topic delivered, and whether a warm hand-off to a patient navigator occurred.

## Data Collection and Navigation Support

Participants were invited to complete a one-page, 10-item form (English or Spanish) that captured age group, gender identity, race/ethnicity (NIH categories), insurance status, zip code, and seven key social determinants of health (food security, housing stability, transportation needs, stress, financial strain, social connections, and intimate partner violence). CHWs keyed responses into the REDCap community EHR within 48–72 h. A JTCC-COE patient navigator reviewed completed screening questionnaires post-event and followed up with participants via their preferred contact method. Navigators then documented every follow-up attempt—phone, text, email, or in-person—and recorded outcomes (appointment scheduled, screening completed, social service referral fulfilled). Screening completion was verified either (a) by the navigator obtaining confirmation from the external provider or (b) by self-report when documentation is unavailable. Despite multiple contact modalities, some participants proved unreachable for outcome confirmation, underscoring a persistent barrier that we address below in the “Discussion” section. Post-event evaluations collected qualitative feedback, and attendance and follow-up outcomes were documented and tracked.

## Community Identification and Engagement Process

### Catchment‐Level Priority Setting

Each January the JTCC-COE team reviews (a) the most recent triennial Community Health Needs Assessment (CHNA) for our three-county catchment area, (b) publicly available New Jersey State Cancer Registry incidence and mortality tables, and (c) state Behavioral Risk Factor Surveillance System data on modifiable cancer-risk behaviors (tobacco use, HPV vaccination, screening uptake). Cancers and behaviors ranked highest on all three sources are flagged as programmatic priorities for the upcoming year.

#### Partner-Driven Site Selection 

Community venues are chosen through two complementary pathways:

1. Community-initiated (~ 45% of sessions). Long-standing community partners—faith-based sites (i.e., churches, temples), Federally Qualified Health Centers, YMCAs, and community-based organizations either (a) invite the JTCC-COE team to bring Paint and Sip to their neighborhood or (b) respond to our outreach communications offering cancer education events. In addition, the quarterly meetings held with the JTCC-COE Community Advisory Council have a standing agenda topic that generates a running list of trusted, non-clinical venues (e.g., churches, senior centers, libraries) where residents already gather.
2. JTCC-COE-initiated (~ 55% of sessions). Using the Community Advisory Council’s rolling “trusted space” list, JTCC-COE staff cold-call or visit locations, often a pastor’s office or a community-based organization’s community room, to share information and ask if an arts-based session will fit the needs of their organization.

#### Topic-Setting Prioritization

When a partner expresses interest, COE staff discuss potential cancer risk and early detection topics of interest and have the partners rate potential topics for the paint and sip activity on *importance to community* and *feasibility within their space/timeframe*. The identified topic becomes the session’s educational focus, and the art motif is co-designed with the artist to match (e.g., pearl of wisdom imagery for colorectal cancer; tree of life for women’s health topics).

#### First-Step Engagement

Prior to scheduling an event, the JTCC-COE Community Engagement Specialist visits the site to (a) meet leadership face-to-face, (b) walk the space to ensure accessibility, and (c) confirm promotion channels (flyers, social media, personal invites). Partners co-brand all materials and collaborate with the JTCC-COE department on participant sign-ups; whereas the JTCC-COE provides supplies, the artist, and patient navigation and follow-up support.

#### Lead Artist and Capacity Building

All sessions were facilitated by the program’s resident artist (FV, co-author), a member of our institution’s Cancer Prevention Precision Control Institute and an artist using paint as a medium with more than 20 years of art experience; he has co-designed the Paint and Sip script (Online Appendix A), models judgment-free facilitation, and shares personal testimony to humanize screening messages. To promote sustainability, he has created a train-the-trainer curriculum—covering acrylic techniques, inclusive language, and timed “drying-time” health dialogues—that prepares CHWs and interested partners to lead future sessions without his direct presence.

## Built-In Flexibility and Rapid-Cycle Adaptation

### Team Structure

COE staff (CHWs, patient navigator, manager) and the lead artist meet for a 20-min “micro-huddle” 48 h before each session to review venue constraints, language needs, and partner requests. Roles are cross-trained such that CHWs can assist with art facilitation, and the artist can co-deliver health content so that tasks can shift on the fly. For example, during a recent Paint and Sip event at a municipal community center, the CHW slated to deliver the breast cancer screening educational mini-lesson was unable to attend at the last minute. The lead artist opened the CHW slide deck on his tablet, followed the speaker note prompts, and wove in a personal family story about timely mammography while a patient navigator fielded specific screening-related questions.

### Plan-Do-Study-Act (PDSA) Loop

After each event, the on-site team records “plusses” and “deltas” in REDCap within 24 h. These notes are then discussed as a standing agenda item during the weekly JTCC-COE virtual department huddle, where the full team reviews the feedback and approves rapid-cycle adjustments. Because the session script is version-controlled, approved edits—such as refining slide-deck language, swapping the art motif, or adjusting the length of the drying-time education break—can be implemented before the next session.

**Figure:**
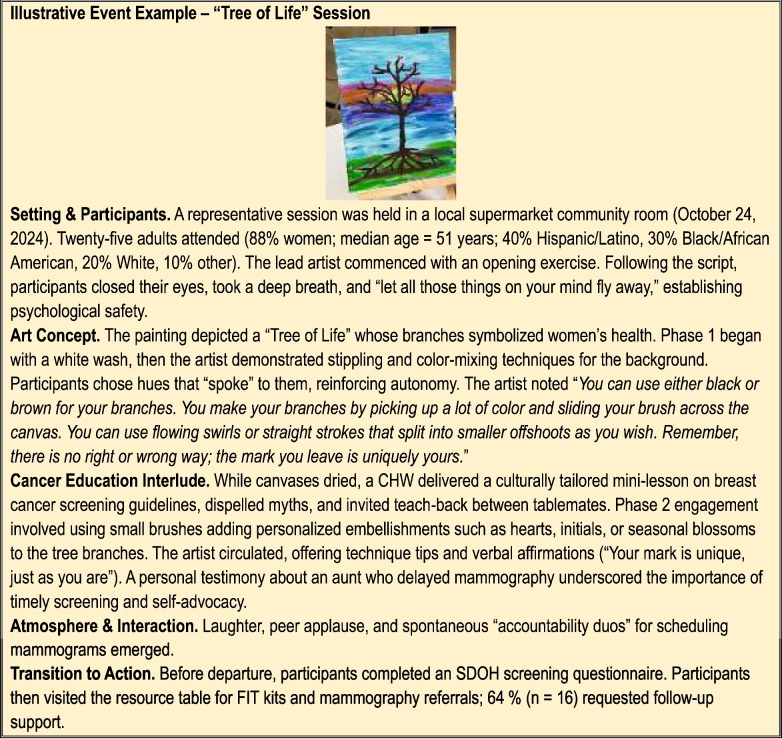
Illustrative event example—“Tree of Life” session

**Figure:**
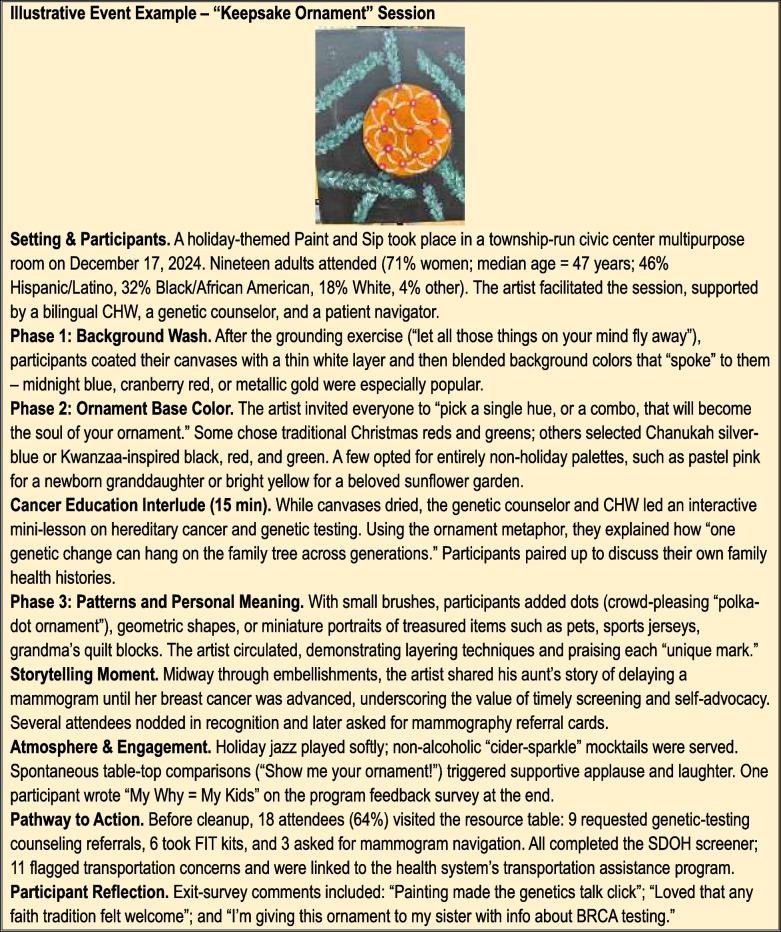
Illustrative event example – “Keepsake Ornament” session

## Results

Since its launch, the Paint and Sip series has been remarkably well-received by community members. Six events have engaged 191 participants across racially and ethnically diverse communities in the Northern New Jersey counties of Bergen, Hudson, and Passaic; 180 (94% completed the SDOH screening questionnaire). Seventy-four percent were women, and ages ranged from 22 to 78 years (median = 52 y; IQR = 43–60). Self-identified race/ethnicity was 41% Hispanic/Latino, 29% Black/African American, 24% White, and 6% other or multiracial. Forty-two percent reported being uninsured or under-insured, 67% reported annual household income < $50,000, and the most common SDOH needs reported were transportation (38%), food security (34%), and housing stability (21%). Attendance averaged 22 participants per session, with many participants returning for subsequent events and bringing friends or family. Feedback has been overwhelmingly positive, with participants describing the events as “uplifting,” “informative,” and “judgment-free.” Several individuals have followed through with cancer screenings and referrals.

- **Screening Status and Navigation**: 82% of attendees (*n* = 157) were not up to date on age-appropriate cancer screenings. Sixty-two percent (*n* = 118) accepted navigation support to social services or cancer screening.
- **Screening Completion**: 26 participants (22% of those who accepted navigation) completed at least one screening within 8 weeks of a program (FIT kits for colorectal cancer screening, genetic counseling and screening, mammography).
- **Engagement**: CHWs reported high levels of participant openness, with many attendees sharing personal stories and discussing stigmatized topics.
- **Qualitative Feedback**: Attendees consistently described the sessions as “uplifting,” “informative,” “judgment-free,” and “a wonderful opportunity to learn” (see Table 1).

**Table 1:** Paint and sip post-event representative quotes

“Thank you so much for the lovely event. Appreciate the painting, screenings, and education!”
“This was a really great event! Fun way to raise awareness and to get screenings in! Kudos to everyone who ran this from the people who checked me in to the person guiding the art activity to the person who did the blood draw! Great job!”
“Very interesting session. Good information, beautiful art class. Staff very professional”
“A very enjoyable and informative afternoon. Thanks so much lots of fun!”
“This is a wonderful opportunity to learn about cancer prevention. I had much fun with the paint & sip activity. The art leader was such a joy to be helping us with our masterpieces”
“I think this class was very helpful and I learned how to take care of my body…it was something new for me, I love it!”

## Discussion

The Paint and Sip series exemplifies how embedding creativity into COE can foster deeper community engagement and build trust around cancer prevention and control topics. The model blends community-based participatory research principles, adult learning theory, and health communication strategies to make health education accessible and emotionally resonant. Early, genuine collaboration—from venue selection to topic—was critical for trust and turnout, reinforcing the value of co-leadership rather than after-the-fact consultation.

As an exemplar, the JTCC-COE Paint and Sip model shows strong early outcomes. While still in its first year of implementation, the program has shown promise as a replicable model for community engagement that bridges creativity and health education in a way that resonates deeply with participants.

The use of art lowered emotional barriers and allowed participants to engage with difficult topics in ways that felt personal and reflective. Participants described feeling empowered and supported in a non-intimidating setting. CHWs play a vital role in reinforcing health messages, dispelling myths, and linking education to actionable follow-up. Furthermore, the SDOH screening questionnaires have identified needs such as transportation and insurance gaps. Patient navigators followed up individually, helping participants access services and schedule screenings. This closed-loop support connects education to action.

## Reflections and Lessons Learned

Implementing the Paint and Sip series through a cancer center COE program has yielded valuable lessons about how creativity can be meaningfully integrated into community health promotion, particularly in the context of cancer prevention and control. As with any community-based initiative, the success of this model has hinged not only on the innovation of the idea itself, but also on its intentional design, collaborative execution, and ongoing responsiveness to community feedback. One of the most important insights gained through this work is the potential of the arts to create a psychologically safe space for learning and dialogue. For many participants, cancer remains a topic associated with fear, stigma, or fatalism. The act of painting—guided, social, and expressive—can provide a gentle entry point into these conversations, helping to lower emotional defenses and shift the tone from clinical to creative, from intimidating to approachable. This reframing can allow participants to engage with difficult topics in ways that feel personal, reflective, and even joyful.

The series also highlighted the importance of interdisciplinary collaboration. Bringing together a local artist, trained CHWs, patient navigators, and community-based organizations creates a model that is both professional and rooted in community. Each stakeholder brings unique expertise: artists contribute to the aesthetic and experiential quality of the events, CHWs ground the sessions in evidence-based messaging, and navigators ensure follow-through on screening and resource linkage. These partnerships require thoughtful coordination but ultimately have the potential to enhance both the experience and impact of the events.

Another key lesson is the value of structure within creativity. While the Paint and Sip model embraces flexibility and fun, its success is also driven by a carefully designed format: the alignment of health themes with painting topics, the use of “drying time” for focused education, and the integration of SDOH screening and navigator follow-up [16]. This balance of art and strategy ensures that participants not only enjoy themselves but creates an event with actionable information and a pathway to care.

Flexibility is also critical. Sessions must adapt to community context, age, culture, and space. CHWs must be nimble and tailor messaging and engagement strategies to participants’ needs. Finally, community engagement is reciprocal. Participants contribute stories, questions, and feedback that shape future programming. Listening to community feedback, such as adding more time for questions and answers, offering events in the evening for working adults, or providing child-friendly options, allows the series to remain responsive and inclusive.

These reflections underscore that integrating creativity into community outreach is not merely a novel tactic. It is a value-based approach that honors culture, fosters connection, and promotes equity. As cancer centers across the country seek to engage communities more deeply and inclusively, models like Paint and Sip series offer a compelling blueprint for what is possible when art and public health work hand in hand. Specifically, our data-anchored yet partner-led approach of combining CHNA findings, state registry trends, Community Advisory Council guidance offers a practical blueprint for cancer centers seeking to engage underserved communities through arts-based education through community outreach and engagement.

## Future Directions for Research and Evaluation

The early success of the JTCC-COE Program Paint and Sip series offers a promising foundation for expanding creative, arts-based strategies in community outreach and engagement. As this model continues to evolve, there are valuable opportunities for future research to more systematically evaluate its reach, effectiveness, and long-term impact on cancer prevention and control outcomes. Future research should evaluate reach, demographic equity, and knowledge or behavior changes. Pre/post assessments could explore learning, stigma reduction, and screening intent. Studies should track the full engagement pathway, from outreach to navigation to screening completion. Emotional and relational aspects, such as trust, perceived safety, and connectedness, should also be examined. Implementation research can assess model fidelity, stakeholder perspectives, and scalability across settings. As arts-based COE gains momentum, building evidence around its impact and equity potential is critical. The Paint and Sip model offers a blueprint for scaling culturally resonant, creative strategies that advance cancer prevention and control in underserved communities.

It is important to note that even with navigator support, structural and psychosocial barriers persisted. Insurance authorization and out-of-pocket concerns have the potential to delay scheduling screenings post event after a participant has indicated their desire to complete a cancer screening. Shift workers may struggle to secure time off, and public transportation gaps have the potential to limit access to imaging centers. Additionally, participants may continue to experience a lingering fear of abnormal results despite counseling from CHW and the patient navigator. Future refinements to this model will include on-site mobile mammography/FIT-kit drop-off, real-time appointment scheduling at events, ride-share vouchers, and a brief text-message reassurance series to reinforce readiness. We also plan to include a quarterly data summit with partners to co-design additional solutions.

## Conclusion

As cancer centers and public health institutions strive to deepen community engagement and reduce disparities in cancer outcomes, innovative approaches are needed. The arts are not an add-on—they are a catalyst for equity, connection, and empowerment. The Paint and Sip model demonstrates how embedding creativity into COE can foster deeper engagement and lay the foundation for sustained cancer prevention efforts.

## Supplementary Information

Below is the link to the electronic supplementary material.

ESM 1 (DOCX 21.6 KB)
