# Supplementary material for: “Paint and Sip for Cancer Prevention”: A Novel Arts-Based Community Engagement Strategy to Advance Cancer Education and Screening in Underserved Individuals
Source: J Cancer Educ. 2025 Jun 28;41(3):451–9. doi: 10.1007/s13187-025-02679-y (PMC13222260; doi:10.1007/s13187-025-02679-y)
Supplement: Supplementary file 1 — (DOCX 21.6 KB) [file 13187_2025_2679_MOESM1_ESM.docx]

**Supplement.** Community “Paint & Sip” Event Protocol

| Item | Specification |
| --- | --- |
| Version / Date | v1.0 / 16 Jun 2025 |
| Intended Use | Supplementary Online Material accompanying the manuscript “Creativity as Catalyst: Leveraging Arts-Based Engagement to Promote Cancer Prevention and Control in Underserved Communities.” |
| Purpose | To provide a step-by-step protocol that enables accurate replication of the Paint-and-Sip intervention used to deliver evidence-based cancer-screening education through an arts-based experience. |
| Target Audience | Adults (≥18 y) from community partner organizations (e.g., faith groups, neighborhood associations, cancer survivor networks). |
| Facilitation Team (minimum) | Lead Facilitator: Trained Art Instructor; Trained Artist  Cancer Screening Educator: Community Health Worker, Patient Navigator, Nurse, or Community Outreach & Engagement (COE) Staff Member  Program Coordinator/Assistant: Session logistics and participant support |
| Session Length | 2 h active program + 30 min clean-up |

1. Materials & Equipment

| Category | Quantity (per participant unless noted) |
| --- | --- |
| Canvas | 1 x 12” x 16” pre-stretched canvas |
| Paint | Student-grade acrylics: white, primary red, yellow, blue, black, brown + optional accent colors |
| Brushes | 1 large flat (~1”) + 1 small round/detail (~2-3 mm) |
| Water Cup | 1 (clear, 12-16 oz) |
| Paper Towels | >4 full-size sheets |
| Palette | Disposable plate or plastic palette |
| Table Coverings | Waterproof sheeting (per table) |
| Easels | Table-top easel or flat workspace |
| Audio Device (optional) | Soft instrumental background music |
| Educational Aids | - 15-min screening slide deck - print brochures and QR codes for screening resources |
| Additional Supplies | Name tags, pens, evaluation forms |

2. Pre-Session Preparation (30 min)

1. **Venue set-up**: Cover tables, place easels/canvases, and arrange supplies at each seat.
2. **Visual examples**: Display exemplar painting at the front.
3. **Educator briefing**: Confirm slide deck load, projector/screen, and resource table placement.
4. **Fidelity check**: Ensure all handouts are current and facilitators have reviewed this protocol.

3. Session Flow & Script

| Segment | Lead | Approx. Time | Key Content & Actions |
| --- | --- | --- | --- |
| A. Welcome & Gratitude | Program Coordinator | 5 min | Introduce JTCC-COE team; thank host organization and participants |
| B. Mindful Opening Exercise | Lead Facilitator | 3 min | Guided breath; script provided (see 3.1) |
| C. Orientation & Objectives | Program Coordinator | 2 min | Explain dual goals: art-making and cancer screening knowledge |
| D. Tool Demonstration | Lead Facilitator | 5 min | Show brushes, water use, palette, emphasize “no right or wrong mark” |
| E. Painting Phase 1 – Background | Lead Facilitator | 20 min | Wet-on-wet technique with white base, followed by participant-chosen background colors. Walk-around coaching. |
| F. Mini-Presentation on Cancer Screening | Educator | 10 min | Slide deck covering breast, cervical, colorectal, lung screening guidelines; local navigation resources |
| G. Painting Phase 2 – Figures & Forms | Lead Facilitator | 20 min | Participants add elements using small brush guided by Lead Facilitator; techniques demonstrated live |
| H. Personal Cancer Story | Lead Facilitator | 5 min | Share scripted narrative to personalize screening importance |
| I. Painting Phase 3 – Embellishments | Lead Facilitator | 15 min | Assist with patterns, highlights, silhouettes; encourage individual creativity |
| J. Reflection & Call-to-Action | Educator | 5 min | Reinforce key screening messages, self-advocacy |
| K. Showcase & Closure | All | 5 min | Group applause, photographs (if consented), invitation to resource table |
| L. Clean-Up | Team | 30 min | Collect brushes, water cups; remove table coverings; restore venue |

3.1 Script Excerpts

Facilitators should adhere to the tone and key phrases below; adapt only for local language or culture.

**Mindful Opening**

“Take a deep breath in… and exhale. Let the thoughts that keep you stuck float away with your breath. You are in a safe space; your presence is enough. Tonight, let every brushstroke come from that inner awareness of who you are.”

**Creativity Norms**

“There is no wrong or right here – only your unique mark. Do not compare your canvas to anyone else’s; they are on their own journey.”

**Cancer Story Excerpt****

“…Maybe if my aunt had received her mammogram on time, she would be painting with us tonight. Screening saves lives. You know your body best—if one provider doesn’t listen, find one who will.”

***this is an example and will change from program to program based upon the individual experience of the person who is sharing*

4. Quality Assurance & Fidelity Monitoring

| Step | Indicator |
| --- | --- |
| Facilitator Training | All facilitators complete a 1hour briefing + observe one live session. |
| Dosage | Session delivered for >90 min painting + >15 min education. |
| Content Coverage | Slide deck completed; personal story told; resource table staffed. |
| Participant Engagement | ≥80% participants remain through reflective close. |
| Immediate Debrief | Session team logs “plusses & deltas” in REDCap within 24 hours; items added to standing agenda of weekly JTCC-COE huddle for rapid-cycle adaptation. |
| Palette | Disposable plate or plastic palette |
| Table Coverings | Waterproof sheeting (per table) |
| Easels | Table-top easel or flat workspace |
| Audio Device (optional) | Soft instrumental background music |
| Educational Aids | - 15-min screening slide deck - print brochures and QR codes for screening resources |
| Additional Supplies | Name tags, pens, evaluation forms |

5. Safety & Ethical Considerations

- COVID-19 precautions per local guidelines.
- Obtain verbal permission before photographing participants or artwork.
- Provide opt-out for sharing personal health stories.
- Ensure accessible venue and non-alcoholic beverage option.

6. Troubleshooting Tips

| Issue | Mitigation |
| --- | --- |
| Paint dries too quickly | Mist canvas lightly with water spray |
| “Muddy” colors | Replace water cups mid-session; remind participants to rinse thoroughly |
| Participant distress during cancer story | Have Educator available for private conversation and referral |
